# Occupational hazards and health cost of women cotton pickers in Pakistani Punjab

**DOI:** 10.1186/s12889-016-3635-3

**Published:** 2016-09-13

**Authors:** Khuda Bakhsh, Naeem Ahmad, M. Asif Kamran, Sarfraz Hassan, Qasir Abbas, Rashed Saeed, M. Sadiq Hashmi

**Affiliations:** 1 Department of Management Sciences, COMSATS Institute of Information Technology, Vehari, Pakistan; 2 Public Health Engineering Department, Vehari, Pakistan; 3 Nuclear Institute for Agriculture and Biology (NIAB), Faisalabad, Pakistan; 4 Institute of Agricultural and Resource Economics, University of Agriculture, Faisalabad, Pakistan; 5 Social Sciences Research Institute, PARC, Faisalabad, Pakistan; 6 Department of Economics, Karakoram International University, Gilgat, Pakistan

**Keywords:** Cotton, Pesticide, Women cotton pickers, Health cost, Pakistan

## Abstract

**Background:**

Farm workers and female cotton pickers are exposed to residual impacts of pesticide use in cotton production, in addition to dust, ultraviolet radiation, etc. Cotton picking causes various health hazards among cotton pickers with varied health cost. A soil bacterium known as *Bacillus thuringiensis* (Bt) is incorporated in cotton seed through genetic modification and it has resistance against certain bollworms of cotton. So it is considered that Bt cotton fields have less pesticide exposure compared to non-Bt cotton fields. This study was designed to examine and compare the impacts and health cost of cotton picking among female cotton pickers working in Bt and non-Bt cotton fields.

**Methods:**

The study used the data collected from Vehari district of Pakistani Punjab. Health hazards and associated health cost of the respondents involved in Bt cotton picking were compared with those who harvested non-Bt cotton. Comparative use of the personal protective measures among those respondents was also examined. Health cost function and its determinants were analyzed using ordinary least square method.

**Results:**

Findings of the study showed that 61 % cotton pickers from Bt cotton households reported one or more health effects of pesticide during picking season whereas this percentage for non-Bt cotton households was 66 %. Health impacts included skin problems, headache, cough, flu/fever, eye irritation and sleeplessness, however, percentage of these health impacts was comparatively higher among non-Bt cotton households. Health cost from exposure to pesticide use in cotton was US$ 5.74 and 2.91 per season for non-Bt cotton and Bt cotton households, respectively. Education, picking in Bt cotton fields and preventive measures were significantly related with health cost.

**Conclusion:**

Cotton pickers working in Bt cotton fields are found to have less occupational health hazards compared to those working in non-Bt cotton fields. Thus generating awareness among cotton pickers for adopting precautionary measures during harvesting and the use of Bt cotton seed can result in a decline in the ill-effects of cotton picking.

## Background

As pesticide is commonly used to control pests, cotton bolls and other parts of cotton contain pesticide residues, its application in cotton production causes various health hazards to human in general and farm workers in particular [1, 2]. Koleva and Schneider [3] estimated an economic loss of US$17 per acre to public health as a result of pesticide use in USA. In Pakistan, the situation can be even the worst as farmers and farm workers are less aware of pesticide externalities and rarely use any precautionary measures. Farmers and farm workers give low priority to health consideration and grossly underestimate health risk of pesticide use [4]. Women are especially vulnerable to pesticide exposure and other pollutants during cotton picking due to their low economic independence in the society. Around 0.1 million women cotton pickers are employed on 1.6 million cotton-growing farms in Pakistan during harvesting season. These women are highly exposed to residual effects of pesticides for a period of 6–8 h daily. Studies show that 74 % female cotton pickers are moderately pesticide-poisoned and the remaining quarter has reached precarious levels of poisoning in Pakistan [5]. The severity of problem is further aggravated due to negligible use of preventive measures among pickers [6].

Occupational exposure to pesticide and its associate impact on human health are well-evident. Studies also show that agriculture workers including cotton pickers experience exposure to pesticides even when they are not directly involved in pesticide use [7–9]. Primary route to exposure of pesticides for cotton pickers include respiratory inhalation and dermal absorption [10, 11]. So cotton pickers are probable to be exposed of both types of routes due to long working hours [12] and inhaling in polluted environment. Khwaja [13] argue that female cotton pickers are more exposed to pesticide use during cotton picking as they work from morning to till sun dust. Female cotton pickers are also exposed to pesticide in the form of inhaling in polluted environment when the adjacent fields are sprayed [14]. Medical studies conducted in cotton growing areas of Pakistan also confirm that women cotton pickers are highly exposed to pesticide residues [15–17]. Women involved in cotton picking report different symptoms, like skin irritation, headache, nausea, gastroenteritis, general weakness, dizziness, vomiting, blisters, fever and stomach pain [13, 17].

In rural areas of Pakistan, women are engaged in different types of farm activities. Some tasks are primarily done by women only. One of them is cotton picking. Women are found picking cotton on family farms while supervised by the elder woman or male head of family. There are also cases when women provide their services for cotton picking to other farms in the same or neighboring village. Owner of the farm usually supervises cotton picking activity on his/her farm. Decision of cotton picking primarily depends on women farm workers. Women not involved in cotton picking perform other farm activities and domestic chores. Cotton picking is considered as a cash job because pickers are able to receive wages at the end of working day in the form of cash or cotton quantity equivalent to cash. Women work in cotton fields for a period 6 to 8 h and cotton picking season normally lasts for 2½ months as a result of multiple pickings.

Considering timing of pesticide use and cotton picking, farmers make their own decision regarding the start of cotton picking. Usually, cotton picking starts after one day of pesticide application in the cotton fields. Women feel different types of health related problems as they are exposed to various pollutants such as inhaling of pesticide fumes, dust, etc. Although regulations exist ensuring training and the use of plant protection equipment by farm workers including pickers, however, such regulations are rarely practiced. Similarly, instructions relating to entering cotton farms after pesticide use are available with the department of agriculture. There are the two most important regulations governing pesticide use in Pakistan. They include the 1971 Agricultural Pesticides Ordinance and the 1973 Agricultural Pesticides Rules. These regulations have provision for the safety of workers handling pesticides and working in farms where pesticides are used. Employers are required not to employ children (aged below 16 years) and elders (aged above 60 years), to ensure training regarding precautions relating to pesticide handlings, to provide protective clothing and respirator or dust filter, not to allow workers without protective clothing to do job, etc. Considering storage of pesticide products, there are missing facilities of proper storage. Pesticides are kept in the sleeping rooms, even in kitchen of households. Moreover, empty containers of pesticides used for carrying water are commonly observed [18] in spite of strictly forbidden reuse of empty containers. All such laws and regulations are not observed/implemented properly, mainly due to weak institutions [19].

Although all age groups of women are engaged in cotton picking, female children in the age of 6 to 8 years, landless poor women are commonly involved at very low wages [6]. Low earnings from cotton picking leave no choice for cotton pickers to spend on health, thereby deteriorating health conditions of cotton pickers.

One way to reduce adverse impacts of pesticide on human health is to reduce pesticide use in cotton production. Integrated pest management, biological control, mechanical methods, bio-pesticides and seed having resistance against cotton pests are some methods. Genetically modified cotton varieties involving *Bacillus thuringiensis* (Bt) gene cotton seed is readily available option to decrease pesticide use in cotton production. It was informally introduced in Pakistan during early 2005 with an aim of declining pesticide use in cotton production. Many studies reveal that Bt cotton seed has substantially declined pesticide use in Pakistan [20–24]. However, effects of the reduced pesticide use on well-being and health of cotton pickers in the form of positive externality of Bt cotton seed have not yet been studied as these studies are relevant to understand economic benefits of using Bt cotton seed. However, social, cultural and economic characteristics are important aspects to understand vulnerabilities of female cotton pickers. There are two types of cotton pests, including sucking and chewing pests. Pesticides are used to control these pests. Bt cotton has resistance against chewing pests i.e. bollworms, so farmers apply pesticides to control sucking pests. Thus, farmers use pesticides on both types of cotton, but cotton pickers working in the fields of Bt cotton can be considered as low exposed to pesticides due to the less use of pesticides against bollworms. On the other hand, farmers growing non-Bt cotton make intensive use of pesticides to control sucking pests and bollworms. So farm workers including cotton pickers may be highly exposed to pesticide residues in non-Bt cotton fields. In such circumstances, the need is to have the scientific evidence on the impact of Bt cotton on the health of cotton pickers. Thus, the present study is designed to fill this information gap. Specific objectives of the study are to identify health hazards due to pesticide exposure and health cost of cotton pickers. The study also examines determinants of health cost of cotton pickers. The remaining article is divided into four sections, namely materials and methods, results, discussion, and conclusions.

## Methods

### Description of cross-sectional study site

The focus of the study was on Punjab province as cotton is mainly planted in this part of the province due to suitable climatic and land fertility conditions. The Vehari district is selected for the study because it has more than 9 % share in area and production of cotton in the Punjab province [25]. Vehari district is situated in the southern part of the Punjab province, 297 km from Lahore, capital of Punjab province. Further, this district is situated between river belt of Sutluj and Chenab rivers. So it possesses highly fertile land suitable for diverse nature of crops, fruits and vegetables.

The data collection was conducted at the end of cotton picking season 2010 from ten villages of Vehari district. Farmers in this district primarily produce cotton, sugarcane, maize and wheat. Pesticide application on cotton usually starts from June and lasts till October whereas cotton picking is expected to begin in September and comes to an end in mid of November. Unfortunately, the data was gathered at the end of cotton picking season. This affected the outcome of our analyses as we were not able to collect information on type of pesticide use and the resultant health impacts on cotton pickers.

### Selection of the participants

Like many other developing countries, farming contributes substantially in the livelihood of farm households. Thus, farming involves all household members to be engaged in different farm activities. Women perform various farm activities including sowing, weeding, hoeing and harvesting. Some farm activities are solely carried out by women and cotton picking is one such activity.

The data collection team, consisting of first four authors, visited and established contact with village leaders in ten villages selected randomly before starting the data collection. This helped to identify fields of Bt and non-Bt cotton in each selected village. With the help of village leaders, a list of households producing Bt and non-Bt cotton households was prepared. The team also made schedule of the data collection during the meetings with the village leaders.

We collected cross-sectional data from 270 randomly selected female cotton pickers from 10 villages. One cotton picker was randomly selected from each randomly selected household from the list. The list included both Bt and non-Bt cotton households. However, Bt cotton seed was commonly used in the selected villages. So our sample size included large number of Bt cotton households (196 number) compared to non-Bt cotton households (74 numbers). The sample size seems enough to understand realistic picture of health impacts and associated cost of cotton picking, based on the experience of other studies conducted in other parts of the world [26–28].

A questionnaire was developed after consulting experts, field workers and cotton pickers and pre-tested questionnaire was used to collect information. In addition to demographics, the questionnaire contained information on knowledge of pesticide use, exposure to pollution during picking, precautionary measures used, health impacts of cotton picking and cost related to health impacts resulting from cotton picking. Since health impacts of cotton picking and costs are based on the experience and recall of the past, the interviewers asked the respondents whether they observed particular symptoms during and or after picking in the previous year. The selected respondents were again asked about the symptoms in the present picking season. Doing this ensured greatly that health impacts reported by the respondents were related to cotton picking.

The questionnaire was prepared in English. The interviewers with excellent speaking and understanding of the local language were hired and trained. Those interviewers translated questions in the local language during interview method. Validity of the questionnaire was conducted before going for final data collection. Pilot testing was done to explore any difficulty during data collection. Accordingly the corrections were made in the questionnaire and data collection method.

Authors with data collection experience supervised the survey. First two authors arranged 2 days training on the questionnaire and consent process to the interviewers for ensuring standardization and good quality of data. It was ensured in the training that the interviewers understood meaning of the questions. The trainers also discussed possible ways of translating each question from English to the local language. All the filled questionnaires were cross-checked by the supervising experts for ensuring consistency and accuracy.

The data collected by the interviewers from the cotton pickers totally depended on the past recalls. The information on the past recall may have biased information which may cause inaccuracy in the estimated results. We considered only those women who were involved in cotton picking continuously.

### Empirical analysis

The collected data was entered, edited and analyzed using descriptive and econometric methods. Descriptive methods included frequency, percentage, mean and median. As Bt cotton is considered to have resistance against certain bollworms of cotton, the use of pesticide may decline. So our sample included two types of farm fields i.e. Bt cotton and non-Bt cotton fields. We found that both types of seeds were used by the farmers in the study area. So we have information relating to female cotton picking on both types of cotton fields. Pollutants existing in the fields during cotton picking remained the same except residual effects of pesticides. So we considered non-Bt cotton fields as comparison group in the present study. The first four authors were involved in data analysis. STATA11 was used to analyze the data.

Pollution related health cost can be accessed through a formal model, considering that pollution causes morbidity. Morbidity is thus related with individual’s welfare i.e. utility. The effects resulting from morbidity include pain and discomfort, loss of productive time, medication cost and expenditures on precautionary actions [26]. Although cotton pickers are not involved in pesticide spraying, they are exposed to residual effects of pesticide, in addition to dust, ultraviolet radiation, long working hours, dehydration, etc., during cotton picking.^1^ So we employ commonly used pesticide exposure model in the present study. Pesticide exposure studies assume that individuals behave in a way to maximize utility, subject to a health production function.

We employed health cost function in the present study. It is similar to a utility maximizing or a health production function [26]. Exposure of women during cotton picking causes a decline in women’s wellbeing as a result of sickness, lost wages and medical expenditures. In the present study, health cost of women cotton pickers considers the cost of illness and preventive measures used, if any. Cost of illness comprises lost productivity and the cost of medical care due to sickness. Productivity loss was estimated by multiplying wages of cotton pickers with work days lost due to sickness. Work days loss included patient and accompanied person. Wage rate prevailing in the villages was used in computing productivity loss in monetary term. Wages for cotton pickers are competitive in villages where cotton is planted. Hiring of labor services for cotton picking in villages is common. We used actual wages for hired cotton pickers whereas the village wage rate was used for family cotton pickers. Costs on preventive actions include defensive expenditures taken during harvesting to minimize health costs. Health cost of cotton pickers does not consider discomfort, pain and suffering due to illness. We build our study on the work of Atreya [26]. The average health costs of cotton pickers are estimated as:

1 $$ \mathit{\mathsf{H}}{\mathit{\mathsf{C}}}_{\mathit{\mathsf{i}}}=\mathit{\mathsf{C}}{\mathit{\mathsf{I}}}_{\mathit{\mathsf{i}}}+\mathit{\mathsf{P}}{\mathit{\mathsf{C}}}_{\mathit{\mathsf{i}}} $$

Where $$ {HC}_i $$ is health cost of i-th respondent in US$ per season due to pesticide exposure, $$ {CI}_i $$ shows cost of illness of the i-th respondent in US$ per season, if any and $$ {PC}_i $$ is the cost (US$/picking season) on preventive actions taken during cotton picking by i-th cotton pickers. Cost of illness includes doctor fee, costs on hospitalization, laboratory and medication, travel cost to and from doctor clinics, opportunity cost of time spent in traveling, dietary expenses during illness, lost work efficiency, and lost workdays of family member in caring the sick person. Costs on preventive actions are costs relating to precautions for reducing pesticide exposure. Such measures can be masks, handkerchiefs, shoes, long-sleeved shirts/pants, glasses, etc. We considered only those averting measures specifically used during cotton picking.

Health cost of cotton pickers can be affected by number of factors. Such factors include socioeconomic characteristics of respondents, duration of cotton picking, nature of farm work, etc. General form of health cost function can be written as

2 $$ \mathit{\mathsf{H}}\mathit{\mathsf{C}}=\mathit{\mathsf{f}}\left[\mathit{\mathsf{S}},\ \mathit{\mathsf{P}\mathsf{M}},\ \mathit{\mathsf{E}}\mathit{\mathsf{X}}\mathit{\mathsf{P}},\ \mathit{\mathsf{I}}\mathit{\mathsf{N}}\mathit{\mathsf{S}}\right) $$

Where $HC$ stands for health cost of cotton pickers, $S$ represents a vector of socioeconomic characteristics relating to health cost, $PM$ is the use of precautionary measures taken during harvesting activity, $EXP$ is the exposure to pesticide and $INS$ are institutional factors affecting health cost, such as access to hospital and other medical facility. We used different functional forms such as linear, semi-log and log-linear. On the bases of our priori expectations and significance of coefficients, we finalized log-linear functional form of health cost of cotton pickers. Empirical model used is given as under:

3 $$ \mathit{\mathsf{lnHC}}={\beta}_{\mathsf{0}}+{\beta}_{\mathsf{1}}\mathit{\mathsf{lnAGE}}+{\beta}_{\mathsf{2}}\mathit{\mathsf{lnEDU}}+{\beta}_{\mathsf{3}}\mathit{\mathsf{B}}\mathit{\mathsf{T}}\_\mathit{\mathsf{NONBT}}+{\beta}_{\mathsf{4}}\mathit{\mathsf{P}}\mathit{\mathsf{C}}+{\beta}_{\mathsf{5}}\mathit{\mathsf{lnDIST}}+\varepsilon $$

Where $$ lnHC $$ is natural log of health cost of cotton pickers (US$/season). $$ lnAGE $$ is taken as independent variable of the respondents (natural log of year) and we expect that this variable would be positively related with health cost as elder cotton pickers are expected to be vulnerable to residual effects. Years of schooling of cotton pickers is taken in log form ($$ lnEDU $$) to examine impacts on health cost as the more educated cotton pickers would be able to avoid adverse effects of cotton picking through employing precautionary measures. Some respondents had no schooling years, we replaced zero values by a very small value before taking log of the variable. $$ BT\_ NONBT $$ is a dummy variable showing that the respondent is involved in cotton picking on Bt cotton fields (1) or non-Bt cotton fields (0). $$ PC $$ shows the effect of preventive measures taken during cotton picking activity and it is taken as one if the respondent uses any precautionary measure, else zero. Access to health facilities is considered to be negatively related with health cost of cotton picking because individuals would be aware of precautionary measures due to continuous consultation with medical staff and they could get treatment immediately. This results in low health cost due to early stage treatment of the disease. To consider the role of access to health facility, we take distance of rural hospital from the village in km ($$ lnDIST) $$. ε is usual error term normally distributed with zero mean and constant variance. β’s are unknown parameters to be estimated. Table 1 shows descriptive statistics of variable used in the present study.

**Table 1:** Definition of variables and econometric results of health cost function

Variables	Unit	Mean (SD)	Coefficients (SE)
Age (lnAGE)	Years	37 (11)	0.51 (0.43)
Education (lnEDU)	Schooling years	2.7 (3.2)	0.38 (0.18)**
Picking on Bt cotton fields (bt_notbt)	1 = Bt cotton fields	0.7 (0.4)	−0.53 (0.32)*
Precautionary measures (PC)	1 = precautionary measures	0.3 (0.5)	1.32 (0.29)***
Distance from hospital (lnDIST)	Km	2.4 (2.2)	−0.32 (0.23)
Constant			2.61 (1.50)***
R^2^			0.10
F value			5.43*

Standard deviations and standard errors in parentheses

* *p* < 0.10

** *p* < 0.05

*** *p* < 0.01

### Ethical considerations and limitation of the study

We prepared a separate information sheet and the consent form. The information sheet contained information on the purpose and confidentiality of data collected and willingness of the individual to participate in the survey. The information sheet was provided to the respondents by the trained interviewers. After getting the written consent from the selected female cotton pickers, questionnaires were filled.

## Results

### Socioeconomic characteristics and vulnerabilities to cotton picking

Cotton picking is solely performed by women in Pakistan. Thus, they are directly exposed to pesticide during cotton harvesting. Socioeconomic characteristics in Table 2 show that out of the total respondents surveyed, 39 % cotton pickers from households growing Bt cotton and 34 % cotton pickers from non-Bt cotton households were between the ages of 14 and 30 years and 37 and 39 % were above 40 years age in the respective households. Further, 72 % female cotton pickers from Bt cotton households were from poor households whereas this percentage was 34 % among non-Bt cotton households. Only 66 % cotton pickers from Bt cotton households were literate and this percentage was 42 % among non-Bt cotton households.^2^

**Table 2:** Socioeconomic characteristic of the respondents and illness reported by the respondents

Characteristics	Number of cotton pickers (%)	
	Bt cotton households	Non-Bt cotton households
Age group (years)		
14–30	67 (34)	29 (39)*
31–40	56 (29)	16 (22)*
Above 40	73 (37)	29 (39)
Education		
Illiterate	67 (34)	43 (58)**
Literate	129 (66)	31 (42)**
Cotton picking experience (years)		
1–5	45 (23)	18 (24)
6–10	38 (19)	16 (22)
11–15	45 (23)	6 (8)**
Above 15	68 (35)	34 (46)**
Household monthly income (US$)		
Below 118	142 (72)	26 (35)**
118–175	45 (23)	42 (57)**
Above 175	9 (5)	6 (8)**
Illness due to cotton picking (No.)		
No effect	77 (39)	25 (34)*
One	51 (26)	21 (28)
Two	39 (20)	15 (20)
Three	12 (6)	6 (8)
Four or above	17 (9)	7 (10)

* *p* < 0.10

** *p* < 0.01

Cotton picking causes various health hazards to cotton pickers. As indicated in Table 2, 26 and 28 % cotton pickers from Bt and non-Bt households respectively fell ill one time during cotton picking. Cotton picking caused illness two times among 20 and 20 %, three times among 6 and 8 % and four times among 9 and 10 % cotton pickers from Bt and non-Bt cotton households respectively. Those reporting no illness were 39 and 34 % from Bt and non-Bt cotton households respectively.

Results show less use of precautionary measures among cotton pickers (Table 3). The sparsely used precautionary measures included gloves (5 and 4 % from Bt and non-Bt cotton households respectively), shoes/socks (12 and 14 % from respective households) and scarf/handkerchief (25 and 28 % from respective households). Above 60 % respondents from both types of households were found using no precautionary measures.

**Table 3:** Precautionary measures used by the respondents

Measures	Number of cotton pickers (%)	
	Bt cotton households	Non-Bt cotton households
No use of any measure	134 (68)	48 (65)
Gloves	9 (5)	3 (4)
Scarf/Handkerchief	48 (25)	21 (28)*
Delaying picking	4 (2)	5 (7)**
Shoes, socks, etc.	23 (12)	10 (14)**

Some respondent reported more than one measures, even no measure so percentage would not be equal to 100

* *p* < 0.10

** *p* < 0.01

The study found number of health problems among cotton pickers (Table 4). Headache (23 % from Bt cotton households and 58 % from non-Bt cotton households), skin related problems (17 and 55 % from respective households), flu/fever (14 and 49 % pickers from Bt and non-Bt cotton households), cough (14 and 38 % from the respective category), eye irritation (5 and 41 % from the respective households) and sleeplessness (12 and 39 % from the respective households) were most commonly revealed effects.

**Table 4:** Health impacts of cotton picking on women

Health impacts	Number of cotton pickers (%)	
	Bt cotton households	Non-Bt cotton households
Headache*	45 (23)	43 (58)***
Eye irritation	9 (5)	30 (41)***
Flue/Fever	27 (14)	36 (49)***
Skin infection/rash	33 (17)	41 (55)***
Asthma attacks	5 (3)	12 (16)**
Shortness of breathing	6 (3)	12 (16)**
Cough	27 (14)	28 (38)***
Dryness of throat	6 (3)	12 (16)***
Nausea/Vomiting	12 (6)	17 (23)***
Dizziness	6 (3)	12 (16)***
Abdominal pain	9 (5)	16 (22)***
Sleeplessness	24 (12)	29 (39)***

Some respondent reported more than one health impacts so percentage would not be equal to 100

* *p* < 0.10

** *p* < 0.05

*** *p* < 0.01

### Precautionary measures and health cost of cotton pickers

**Fig. 1:**
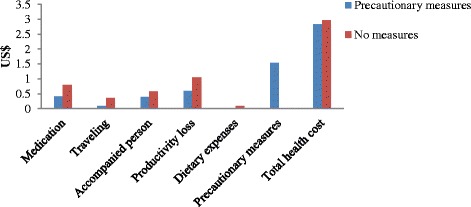
Components of health cost of cotton pickers from Bt cotton households

**Fig. 2:**
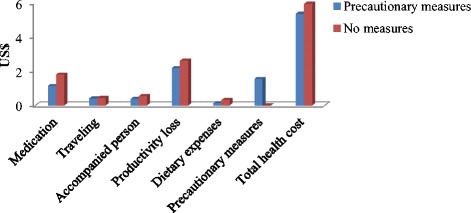
Components of health cost of pickers in non-Bt cotton fields

A total of 35 % cotton pickers from non-Bt cotton households used one or more than one precautionary measures while picking cotton. This percentage is relatively low among cotton pickers from Bt cotton households. We examined the relationship between health cost of cotton picking and precautionary measures used by the pickers. We find that the respondents from Bt cotton households and using precautionary measures have less health cost (US$ 2.84) compared to those using no precautionary measures (US$ 2.96). Although productivity loss is lower, cotton pickers using precautionary measures have to incur cost of preventive measures at the rate of US$ 1.54 per season. The same relationship is found among cotton pickers from non-Bt cotton households (Figs. 1 and 2). Further, the direction of impact of precautionary measures is same in both types of the respondents. However, the health cost and its components are on higher side for non-Bt cotton households. Further, health cost of Bt cotton households with precautionary measures is statistically significant compared to that of non-Bt cotton households with precautionary measures.

### Knowledge of pesticide use

Considering knowledge of cotton pickers relating to pesticide use in the cotton fields, only 18 and 22 % women from Bt and non-Bt cotton households respectively have knowledge of pesticide use in the cotton fields. However, such women except 1 % have no information on the name of pesticide. Women reporting temporary nature of health problems resulting during cotton picking were fewer among Bt cotton households compared to non-Bt cotton households. Approximately all cotton pickers reported to bring the picked cotton from fields to farm house (Table 5).

**Table 5:** Knowledge of pesticide use on cotton fields among cotton pickers

Health impacts	Number of cotton pickers (%)	
	Bt cotton fields	Non-Bt cotton fields
Do you know when pesticide applied to the field?	35 (18)	16 (22)
Do you know the name of pesticide causing ill effect?	2 (1.02)	1 (1.35)
Whether health effect was of temporary in nature?	112 (57)	47 (64)
Did you carry picked cotton from field to farm house?	192 (98)	74 (100)

### Health cost of cotton pickers and its determinants

The present study considered the short-term picking-induced impacts on health of cotton pickers. We considered those cotton pickers who suffered from exposure to pesticide and other factors during cotton picking activity and consulted a doctor. Among components of health cost, productivity loss is huge (US$ 0.89 and 2.47 for Bt and non-Bt cotton households, respectively). Respective mean medication cost of such respondents is found to be US$0.67 and 1.58 per season. Precautionary measures component included costs of gloves, shoes/socks, delayed picking, masks and scarf/handkerchief. Average cost of this component is US$ 0.49 and 0.55 per season for Bt and non-Bt cotton households. Considering mean health cost of cotton pickers, it is US$ 2.91 and 5.74 per season for Bt and non-Bt cotton households, respectively (Tables 6 and 7).

**Table 6:** Health cost of women cotton pickers from non-Bt cotton households (US$/season)

Cost items	Percentiles						Mean
	25 %	50 %	75 %	90 %	95 %	99 %	
Medication	0	0.62	2.73	4.14	5.53	6.76	1.58
Traveling	0	0.24	0.71	0.94	1.76	2.35	0.42
Accompanied person	0	0.41	0.88	0.94	1.41	2.94	0.49
Productivity loss	0	0.71	4.12	7.35	9.41	11.53	2.47
Dietary expenses	0	0.00	0.00	0.35	0.82	0.24	0.24
Precautionary measures	0	0.00	1.18	1.74	2.71	2.94	0.55
Total health cost	0	3.91	9.59	13.71	15.95	20.88	5.74

**Table 7:** Health cost of women cotton pickers from Bt cotton households (US$/season)

Cost items	Percentiles						Mean
	25 %	50 %	75 %	90 %	95 %	99 %	
Medication	0	0.35	1.18	1.78	2.13	4.14	0.67
Traveling	0	0.12	0.35	0.82	1.06	2.35	0.28
Accompanied person	0	0.41	0.88	1.41	2.06	3.00	0.52
Productivity loss	0	0.53	1.50	2.35	2.94	5.06	0.89
Dietary expenses	0	0.00	0.00	0.00	0.59	2.94	0.07
Precautionary measures	0	0.00	0.71	1.53	2.71	3.88	0.49
Total health cost	0	2.00	4.75	7.06	8.38	13.00	2.91

As health cost of cotton pickers are highly skewed, so we also present results in percentiles (Tables 6 and 7). The median of medication of cotton pickers from Bt and non-Bt cotton households (the 50th percentile) is US$ 0.35 and 0.62 per season, respectively. The respective median of the 90th percentile is US$ 1.78 and 4.14 per season. Median of productivity loss (the 90th percentile) of cotton pickers from Bt and non-Bt cotton households is US$ 2.35 and 7.35, respectively. As expected, median of health cost of cotton pickers (each percentile) is highly different between Bt and non-Bt cotton households.

Double log model is used to examine impacts of factors affecting health cost of the respondents (Table 1). Our results show that overall regression model is statistically significant as p-value for F-test is very low. As a priori, we expected a negative impact of education on health cost of cotton pickers. However, coefficient of education variable is positive and highly significant, rejecting a priori expectation. Age variable was considered as a proxy for the extent of exposure to pesticide as people becomes more susceptible with the increase in age. Coefficient of this variable is positive but non-significant.

Dummy variable for the respondents from Bt cotton households shows that the respondents from Bt cotton households have lower cost compared to those from non-Bt cotton households. Dummy variable for using precautionary measures has positive effect on health cost and coefficient is statistically different from zero. The value of variable used for distance from hospital is negative but statistically non-significant (Table 1).

## Discussion

Our findings of the study show that a few schooling years among cotton pickers is an indication that women pickers have little access to education due to financial or other constraints. Thus low literacy implies that cotton pickers may have less awareness regarding external effects of cotton picking. Since instructions on pesticide containers/bottles are written in English and Urdu, women with low/no literacy would not be able to read instructions or even understand symbols of poison and danger level on containers/bottles. Further, small landholdings with very low income dominate in the study area, so women have to start farming activities in their early ages to provide financial support to family. The probability of suffering from various diseases may increase in the long-run because such women would be exposed to adverse effects of cotton picking in the presence of pesticide fumes, dust, ultraviolet radiation, etc. Further, aged women develop less immunity against adverse impacts of cotton picking as well.

We were expecting that cotton pickers may have little or no knowledge about the timings and types of pesticide used in cotton field. Findings of the study support our priori expectation. Women cotton pickers in the study area are found to have little knowledge of pesticide sprayed in the cotton fields before cotton picking. Further, it also shows that cotton pickers from both types of households are exposed to pesticides but cotton pickers in Bt cotton fields are less exposed as a result of pesticide use against sucking pests only. A good percentage of women pickers from both types of households report that health effects of cotton picking especially due to residual effects of pesticide are temporary. Moreover, exposure to ill effects of cotton picking increases as women are also responsible to bring the picked cotton from cotton fields to farm house.

Cotton picking can cause chronic and short-term health impacts. In the present study, our focus was to find out short-term impacts of cotton picking because chronic health problems were difficult to be identified on information based on the past recall. Self-reported symptoms can be categorized into chronic and acute symptoms. Asthma, shortness of breathing, abdominal pain and sleeplessness can be considered as chronic health problems whereas headache, vomiting, dizziness, eye irritation, skin infection, etc. are acute health symptoms. In the present study, women cotton pickers complain of different short-term health impacts resulting from cotton picking. These include dizziness, headache, skin related issues, etc. These statistics also shows severity of picking activity and its impacts on human health. Comparing findings of Tables 2 and 4, we find that the difference of reported illness between Bt and non-Bt households is very small but symptoms mentioned by cotton pickers are on higher side among non-Bt cotton households. This may be due to the presence of huge pesticide residues in non-Bt cotton fields as Bt cotton growing farmers have to employ larger quantity of pesticides to control sucking and chewing pests. Thus cotton pickers in non-Bt cotton fields reported more symptoms relating to cotton picking. Atreya [26] and Shetty et al. [29] find most commonly symptoms including headache, weakness, dizziness, fever, blurred vision, and nausea/vomiting among farm workers in India. Rizwan et al. [16] also find such type of symptoms among cotton pickers. They also show that exposure of cotton pickers causes an increase in hormonal level among women pickers after harvesting season. One other study also report that 10,000 farmers including women are poisoned annually due to exposure to pesticides in Pakistan [30]. The diseases reported by the previous studies [16, 26, 29] are the same health outcomes reported in the present study. It is also evident that few cotton pickers from Bt cotton households report health impacts of cotton picking as compared to non-Bt cotton households. Non-Bt cotton involves huge amount of pesticide use to control cotton pests including sucking and bollworms. Pesticide residues are major pollutant during cotton picking and we assume that other pollutants remain at the same level for both types of farms. This may be the reason that cotton pickers working on non-Bt cotton farms suffer more from cotton picking. However, the reported diseases by the respondents can also be due to factors other than cotton picking. Healthy workers can be less vulnerable to cotton picking than those who have already certain health problems. Dasgupta et al. [31] point out that blood testing is more appropriate because self-reported symptoms are weak indicators of health impacts. But blood sampling at field level in the present study was highly difficult in the absence of finance and medical personnel.

Although personal protective measures are considered to reduce symptoms, exposure to pesticide and other pollutants during picking remains there. We also focus on the type and use of personal protective measures during cotton picking. Most cotton pickers were found using their normal clothes and approximately 30 % respondents reported wearing some protective measures. This little use of protective measures puts lives of cotton pickers at risk. They had perception of no health hazards associated with exposure to pesticides and considered cotton picking as a normal and safe working activity. Therefore, adoption of preventive measures among cotton pickers is minimal. However, we find a statistically significant relationship between personal protective measures and health cost among Bt and non-Bt cotton households. Low health cost of Bt cotton households with precautionary measures is an indication of benefits associated with personal protective equipments to be used during picking. Farm workers use precautionary measures when they see cost associated with exposure to pesticide and resultant pesticide-related illness symptoms and associated costs [32]. However, the effects of exposure are not visible in short-run and workers have to bear huge cumulative effects in the long run [33]. Khan et al. [34] indicate that most farmers (52 %) are of the view that the risk from pesticide use is low, some others consider no risk from exposure to pesticide.

Findings of the present study show that health cost of cotton pickers is low when protective measures are used. However, health cost of pickers from Bt cotton households with protective measures is far less compared to their counterparts. This may be due to the fact that Bt cotton involves less use of pesticide so Bt cotton farms have relatively few residual effects of pesticide compared to non-Bt cotton. One other aspect is absorption of pesticide and other chemicals in protective measures. In such case where protective measures need to be replaced or properly washed otherwise wearing protective measures becomes worse than not wearing any protective measures. However, the present study lacks this information and this is the future research area to be explored in details.

There are different factors affecting health cost of cotton pickers. Productivity loss constitutes large share of total health cost. However, this share is far higher among cotton pickers belonging to non-Bt cotton households. Similarly, the respondents from non-Bt cotton households had to spend comparatively higher amount on medication and precautionary measures. Nevertheless Athukorala et al. [2] argue that precautionary expenditures are important determinant of medical cost of farm workers but in our study, productivity loss is the major contributor of health cost of cotton pickers. Traveling cost is US$ 0.31, indicating availability of medical facility at far distant location. Access to medical facility in the rural areas would reduce additional cost of travelling when one gets some health problem. Women will particularly get benefits from local health centers.

Median and mean health cost is too high for women workers whose daily wages are around US$ 2.94/40 kg. It shows that women cotton pickers are at high risk due to pesticide residual effects as their earnings decline substantially. Shetty et al. [35] find health cost of farmers and farm workers in the range of US$ 2.13 to 10.64 per season in India. Devi [36] estimated US$ 3 per month as welfare loss to pesticide applicator in India. Our estimate of health cost is far below from other studies [35, 36] because those studies consider annual cost and or long-term health impacts.

Findings of the study would have been more interesting in the presence of data before adoption of Bt cotton. Unfortunately, this type of data was not available. Information on risk factors other than pesticides is not considered in the present study. Further, having women not involved in cotton picking would have been a good comparison group. Unfortunately the present study lacks this group. Authors considered only those women who were continuously involved in cotton picking. It means such women were highly exposed to ill effects of cotton picking. Ignoring others involved for a few days in picking can result in bias selection. Since we are interested to examine the impact of Bt cotton on health cost of cotton pickers, we did not select all types of cotton pickers. However, we suggest considering other risk factors and selection of women cotton pickers based on exposure to harvesting and those not involved in harvesting in future research. Further, health cost resulting from exposure to pesticide residual effects was estimated for a single cotton season in the present study. Hence, the estimated health cost may be treated cautionary as the estimates might have been underestimated.

The present study has limitation of no reported symptoms in the population during off-season, or when population is less/unexposed to pesticides. Having such types of respondents would further provide insights on exposure to harvesting of cotton. Duration of cotton picking may vary among women as women picking for a few days would have less exposure to pesticides and other risk factors.

## Conclusions

Exposure to pesticide residues has a significant negative effect on health of women cotton pickers. Using any type of precautionary measures during cotton picking is negligible in the study area. This can be attributed to low literacy and lack of institutional support for training and awareness. Women cotton pickers should also be educated about the importance (in terms of disease treatment and long-run health costs) of using safety precautions while working in cotton fields. The government or extension department may provide protective masks at highly subsidized prices to the female workers working either in cotton picking activities or in other crops/vegetables. Doing so would help reduce health problems of cotton pickers. Broadening net of health facilities in rural areas can decline health cost of those suffering from exposure to pesticides and reduce long run cumulative irreparable health impacts.

Findings of the study show extra economic burden on women cotton pickers who are already vulnerable in the rural setting. In the nutshell, the estimated health cost in this study could be used by policy makers as a justification to start programs focusing on proper handling of pesticide use and safety measures.

## Footnotes

1 Authors acknowledge viewpoint of reviewers that female cotton pickers are exposed to diverse nature of pollutants.

2 Here literate means those who can read and write a few words.
